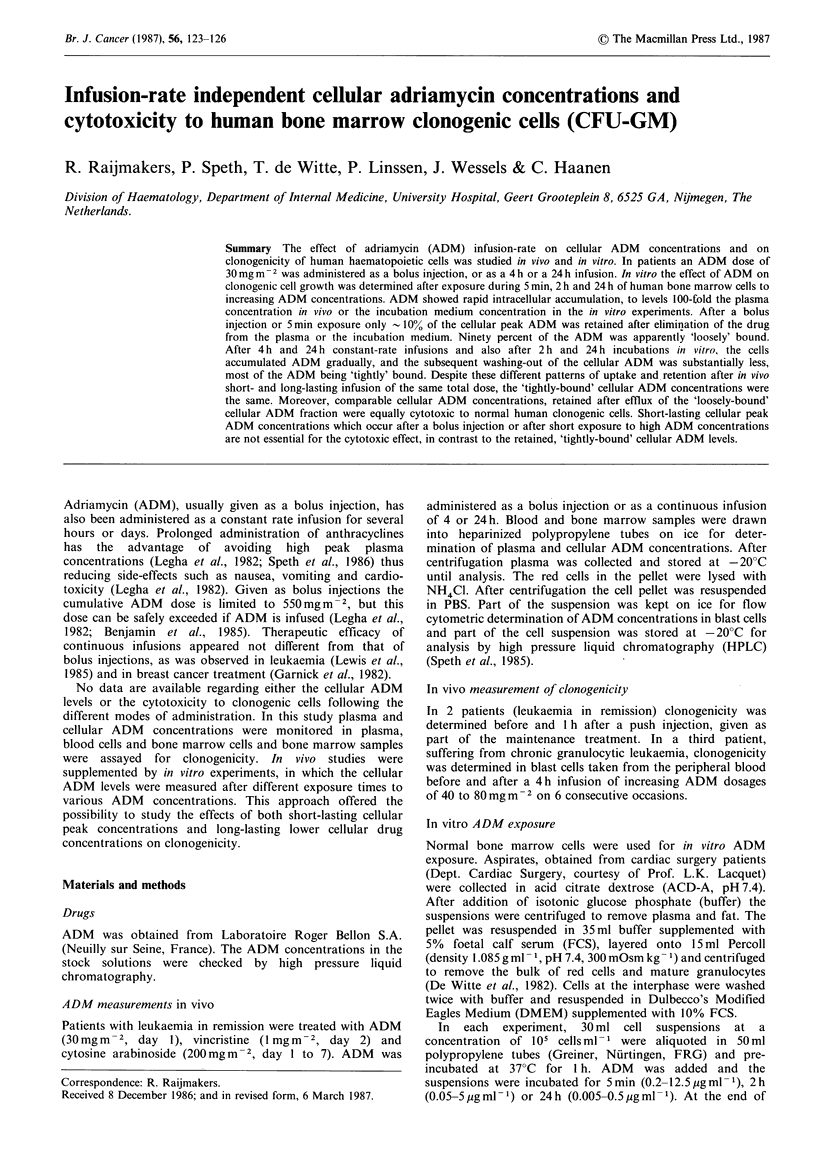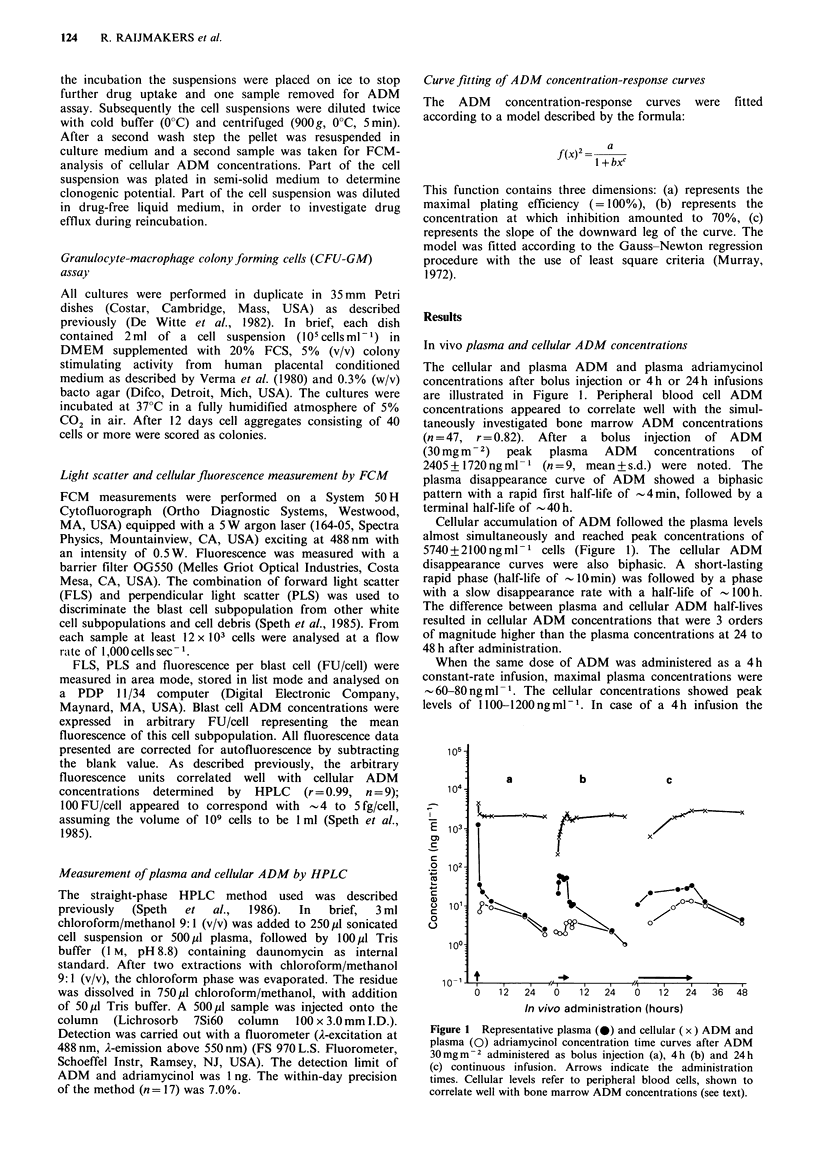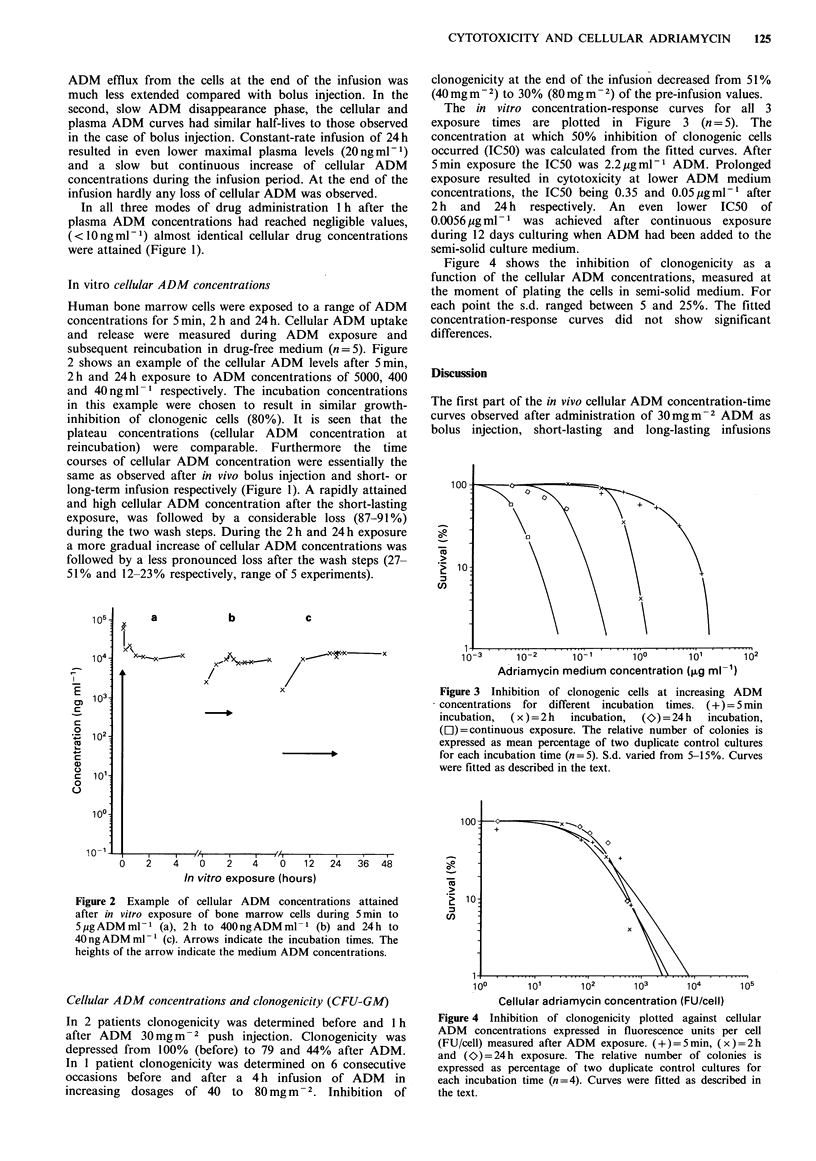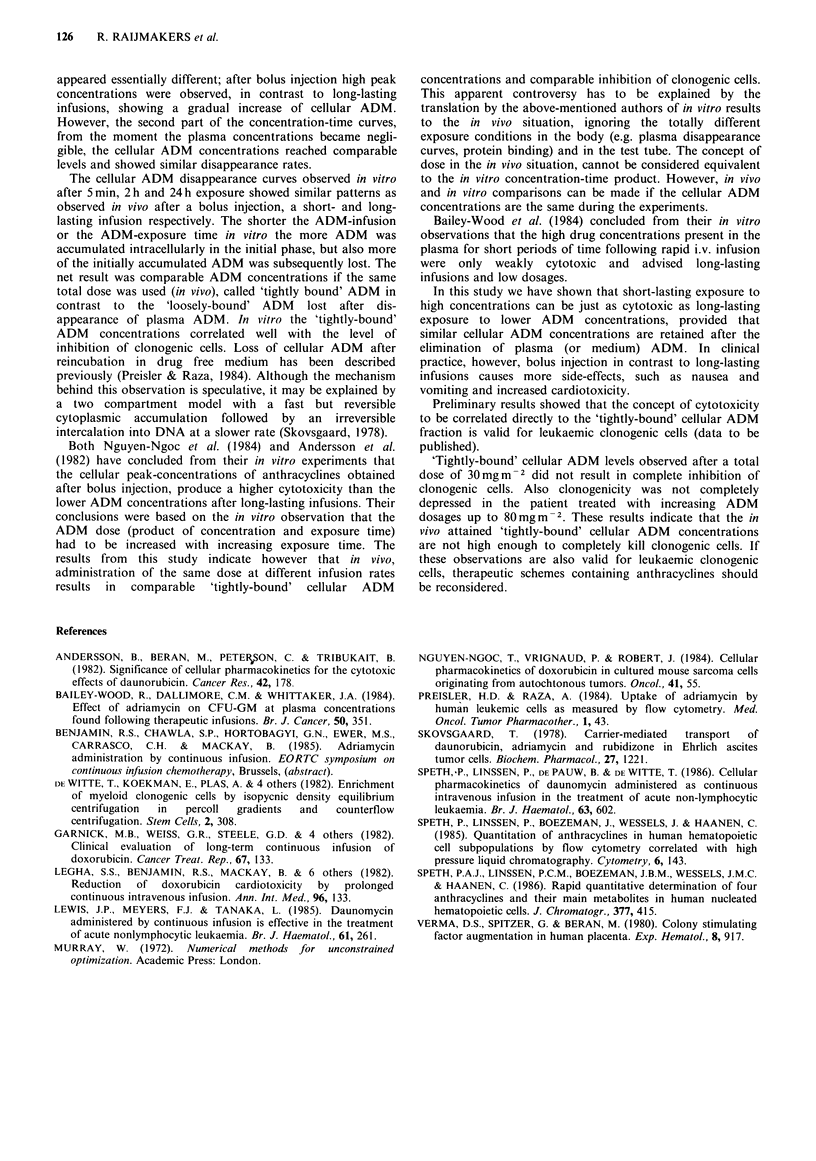# Infusion-rate independent cellular adriamycin concentrations and cytotoxicity to human bone marrow clonogenic cells (CFU-GM).

**DOI:** 10.1038/bjc.1987.168

**Published:** 1987-08

**Authors:** R. Raijmakers, P. Speth, T. de Witte, P. Linssen, J. Wessels, C. Haanen

**Affiliations:** Department of Internal Medicine, University Hospital, Nijmegen, The Netherlands.

## Abstract

The effect of adriamycin (ADM) infusion-rate on cellular ADM concentrations and on clonogenicity of human haematopoietic cells was studied in vivo and in vitro. In patients an ADM dose of 30 mg m-2 was administered as a bolus injection, or as a 4 h or a 24 h infusion. In vitro the effect of ADM on clonogenic cell growth was determined after exposure during 5 min, 2 h and 24 h of human bone marrow cells to increasing ADM concentrations. ADM showed rapid intracellular accumulation, to levels 100-fold the plasma concentration in vivo or the incubation medium concentration in the in vitro experiments. After a bolus injection or 5 min exposure only approximately 10% of the cellular peak ADM was retained after elimination of the drug from the plasma or the incubation medium. Ninety percent of the ADM was apparently 'loosely' bound. After 4 h and 24 h constant-rate infusions and also after 2 h and 24 h incubations in vitro, the cells accumulated ADM gradually, and the subsequent washing-out of the cellular ADM was substantially less, most of the ADM being 'tightly' bound. Despite these different patterns of uptake and retention after in vivo short- and long-lasting infusion of the same total dose, the 'tightly-bound' cellular ADM concentrations were the same. Moreover, comparable cellular ADM concentrations, retained after efflux of the 'loosely-bound' cellular ADM fraction were equally cytotoxic to normal human clonogenic cells. Short-lasting cellular peak ADM concentrations which occur after a bolus injection or after short exposure to high ADM concentrations are not essential for the cytotoxic effect, in contrast to the retained, 'tightly-bound' cellular ADM levels.


Br. J. Cancer (1987), 56, 123–126 © The Macmillan Press Ltd., 1987

### Infusion-rate independent cellular adriamycin concentrations and cytotoxicity to human bone marrow clonogenic cells (CFU-GM)

R. Raijmakers, P. Speth, T. de Witte, P. Linssen, J. Wessels & C. Haanen

*Division of Haematology, Department of Internal Medicine, University Hospital, Geert Grooteplein 8, 6525 GA, Nijmegen, The Netherlands.*

**Summary** The effect of adriamycin (ADM) infusion-rate on cellular ADM concentrations and on clonogenicity of human haematopoietic cells was studied *in vivo* and *in vitro*. In patients an ADM dose of $30\,mg\,m^{-2}$ was administered as a bolus injection, or as a 4h or a 24h infusion. *In vitro* the effect of ADM on clonogenic cell growth was determined after exposure during 5 min, 2 h and 24 h of human bone marrow cells to increasing ADM concentrations. ADM showed rapid intracellular accumulation, to levels 100-fold the plasma concentration *in vivo* or the incubation medium concentration in the *in vitro* experiments. After a bolus injection or 5 min exposure only ~10% of the cellular peak ADM was retained after elimination of the drug from the plasma or the incubation medium. Ninety percent of the ADM was apparently 'loosely' bound. After 4h and 24h constant-rate infusions and also after 2h and 24h incubations *in vitro*, the cells accumulated ADM gradually, and the subsequent washing-out of the cellular ADM was substantially less, most of the ADM being 'tightly' bound. Despite these different patterns of uptake and retention after *in vivo* short- and long-lasting infusion of the same total dose, the 'tightly-bound' cellular ADM concentrations were the same. Moreover, comparable cellular ADM concentrations, retained after efflux of the 'loosely-bound' cellular ADM fraction were equally cytotoxic to normal human clonogenic cells. Short-lasting cellular peak ADM concentrations which occur after a bolus injection or after short exposure to high ADM concentrations are not essential for the cytotoxic effect, in contrast to the retained, 'tightly-bound' cellular ADM levels.

Adriamycin (ADM), usually given as a bolus injection, has also been administered as a constant rate infusion for several hours or days. Prolonged administration of anthracyclines has the advantage of avoiding high peak plasma concentrations (Legha *et al.*, 1982; Speth *et al.*, 1986) thus reducing side-effects such as nausea, vomiting and cardiotoxicity (Legha *et al.*, 1982). Given as bolus injections the cumulative ADM dose is limited to $550\,mg\,m^{-2}$, but this dose can be safely exceeded if ADM is infused (Legha *et al.*, 1982; Benjamin *et al.*, 1985). Therapeutic efficacy of continuous infusions appeared not different from that of bolus injections, as was observed in leukaemia (Lewis *et al.*, 1985) and in breast cancer treatment (Garnick *et al.*, 1982).

No data are available regarding either the cellular ADM levels or the cytotoxicity to clonogenic cells following the different modes of administration. In this study plasma and cellular ADM concentrations were monitored in plasma, blood cells and bone marrow cells and bone marrow samples were assayed for clonogenicity. *In vivo* studies were supplemented by *in vitro* experiments, in which the cellular ADM levels were measured after different exposure times to various ADM concentrations. This approach offered the possibility to study the effects of both short-lasting cellular peak concentrations and long-lasting lower cellular drug concentrations on clonogenicity.

### Materials and methods

### *Drugs*

ADM was obtained from Laboratoire Roger Bellon S.A. (Neuilly sur Seine, France). The ADM concentrations in the stock solutions were checked by high pressure liquid chromatography.

### *ADM measurements* in vivo

Patients with leukaemia in remission were treated with ADM ($30\,mg\,m^{-2}$, day 1), vincristine ($1\,mg\,m^{-2}$, day 2) and cytosine arabinoside ($200\,mg\,m^{-2}$, day 1 to 7). ADM was administered as a bolus injection or as a continuous infusion of 4 or 24h. Blood and bone marrow samples were drawn into heparinized polypropylene tubes on ice for determination of plasma and cellular ADM concentrations. After centrifugation plasma was collected and stored at −20°C until analysis. The red cells in the pellet were lysed with $NH_4Cl$. After centrifugation the cell pellet was resuspended in PBS. Part of the suspension was kept on ice for flow cytometric determination of ADM concentrations in blast cells and part of the cell suspension was stored at −20°C for analysis by high pressure liquid chromatography (HPLC) (Speth *et al.*, 1985).

### In vivo *measurement of clonogenicity*

In 2 patients (leukaemia in remission) clonogenicity was determined before and 1 h after a push injection, given as part of the maintenance treatment. In a third patient, suffering from chronic granulocytic leukaemia, clonogenicity was determined in blast cells taken from the peripheral blood before and after a 4h infusion of increasing ADM dosages of 40 to $80\,mg\,m^{-2}$ on 6 consecutive occasions.

### In vitro *ADM exposure*

Normal bone marrow cells were used for *in vitro* ADM exposure. Aspirates, obtained from cardiac surgery patients (Dept. Cardiac Surgery, courtesy of Prof. L.K. Lacquet) were collected in acid citrate dextrose (ACD-A, pH 7.4). After addition of isotonic glucose phosphate (buffer) the suspensions were centrifuged to remove plasma and fat. The pellet was resuspended in 35 ml buffer supplemented with 5% foetal calf serum (FCS), layered onto 15 ml Percoll (density $1.085\,g\,ml^{-1}$, pH 7.4, $300\,mOsm\,kg^{-1}$) and centrifuged to remove the bulk of red cells and mature granulocytes (De Witte *et al.*, 1982). Cells at the interphase were washed twice with buffer and resuspended in Dulbecco's Modified Eagles Medium (DMEM) supplemented with 10% FCS.

In each experiment, 30 ml cell suspensions at a concentration of $10^5$ $cells\,ml^{-1}$ were aliquoted in 50 ml polypropylene tubes (Greiner, Nürtingen, FRG) and pre-incubated at 37°C for 1 h. ADM was added and the suspensions were incubated for 5 min ($0.2$–$12.5\,\mu g\,ml^{-1}$), 2 h ($0.05$–$5\,\mu g\,ml^{-1}$) or 24 h ($0.005$–$0.5\,\mu g\,ml^{-1}$). At the end of

Correspondence: R. Raijmakers.
Received 8 December 1986; and in revised form, 6 March 1987.

the incubation the suspensions were placed on ice to stop further drug uptake and one sample removed for ADM assay. Subsequently the cell suspensions were diluted twice with cold buffer (0°C) and centrifuged (900g, 0°C, 5 min). After a second wash step the pellet was resuspended in culture medium and a second sample was taken for FCM-analysis of cellular ADM concentrations. Part of the cell suspension was plated in semi-solid medium to determine clonogenic potential. Part of the cell suspension was diluted in drug-free liquid medium, in order to investigate drug efflux during reincubation.

*Granulocyte-macrophage colony forming cells (CFU-GM) assay*

All cultures were performed in duplicate in 35 mm Petri dishes (Costar, Cambridge, Mass, USA) as described previously (De Witte *et al.*, 1982). In brief, each dish contained 2 ml of a cell suspension ($10^5$ cells $ml^{-1}$) in DMEM supplemented with 20% FCS, 5% (v/v) colony stimulating activity from human placental conditioned medium as described by Verma *et al.* (1980) and 0.3% (w/v) bacto agar (Difco, Detroit, Mich, USA). The cultures were incubated at 37°C in a fully humidified atmosphere of 5% $CO_2$ in air. After 12 days cell aggregates consisting of 40 cells or more were scored as colonies.

*Light scatter and cellular fluorescence measurement by FCM*

FCM measurements were performed on a System 50 H Cytofluorograph (Ortho Diagnostic Systems, Westwood, MA, USA) equipped with a 5 W argon laser (164-05, Spectra Physics, Mountainview, CA, USA) exciting at 488 nm with an intensity of 0.5 W. Fluorescence was measured with a barrier filter OG550 (Melles Griot Optical Industries, Costa Mesa, CA, USA). The combination of forward light scatter (FLS) and perpendicular light scatter (PLS) was used to discriminate the blast cell subpopulation from other white cell subpopulations and cell debris (Speth *et al.*, 1985). From each sample at least $12 \times 10^3$ cells were analysed at a flow rate of 1,000 cells $sec^{-1}$.

FLS, PLS and fluorescence per blast cell (FU/cell) were measured in area mode, stored in list mode and analysed on a PDP 11/34 computer (Digital Electronic Company, Maynard, MA, USA). Blast cell ADM concentrations were expressed in arbitrary FU/cell representing the mean fluorescence of this cell subpopulation. All fluorescence data presented are corrected for autofluorescence by subtracting the blank value. As described previously, the arbitrary fluorescence units correlated well with cellular ADM concentrations determined by HPLC ($r=0.99$, $n=9$); 100 FU/cell appeared to correspond with ~4 to 5 fg/cell, assuming the volume of $10^9$ cells to be 1 ml (Speth *et al.*, 1985).

*Measurement of plasma and cellular ADM by HPLC*

The straight-phase HPLC method used was described previously (Speth *et al.*, 1986). In brief, 3 ml chloroform/methanol 9:1 (v/v) was added to 250 $\mu$l sonicated cell suspension or 500 $\mu$l plasma, followed by 100 $\mu$l Tris buffer (1 M, pH 8.8) containing daunomycin as internal standard. After two extractions with chloroform/methanol 9:1 (v/v), the chloroform phase was evaporated. The residue was dissolved in 750 $\mu$l chloroform/methanol, with addition of 50 $\mu$l Tris buffer. A 500 $\mu$l sample was injected onto the column (Lichrosorb 7Si60 column 100 x 3.0 mm I.D.). Detection was carried out with a fluorometer ($\lambda$-excitation at 488 nm, $\lambda$-emission above 550 nm) (FS 970 L.S. Fluorometer, Schoeffel Instr, Ramsey, NJ, USA). The detection limit of ADM and adriamycinol was 1 ng. The within-day precision of the method ($n=17$) was 7.0%.

*Curve fitting of ADM concentration-response curves*

The ADM concentration-response curves were fitted according to a model described by the formula:

$$f(x)^2 = \frac{a}{1+bx^c}$$

This function contains three dimensions: (a) represents the maximal plating efficiency (=100%), (b) represents the concentration at which inhibition amounted to 70%, (c) represents the slope of the downward leg of the curve. The model was fitted according to the Gauss–Newton regression procedure with the use of least square criteria (Murray, 1972).

### Results

In vivo *plasma and cellular ADM concentrations*

The cellular and plasma ADM and plasma adriamycinol concentrations after bolus injection or 4 h or 24 h infusions are illustrated in Figure 1. Peripheral blood cell ADM concentrations appeared to correlate well with the simultaneously investigated bone marrow ADM concentrations ($n=47$, $r=0.82$). After a bolus injection of ADM (30 mg $m^{-2}$) peak plasma ADM concentrations of $2405 \pm 1720$ ng $ml^{-1}$ ($n=9$, mean $\pm$ s.d.) were noted. The plasma disappearance curve of ADM showed a biphasic pattern with a rapid first half-life of ~4 min, followed by a terminal half-life of ~40 h.

Cellular accumulation of ADM followed the plasma levels almost simultaneously and reached peak concentrations of $5740 \pm 2100$ ng $ml^{-1}$ cells (Figure 1). The cellular ADM disappearance curves were also biphasic. A short-lasting rapid phase (half-life of ~10 min) was followed by a phase with a slow disappearance rate with a half-life of ~100 h. The difference between plasma and cellular ADM half-lives resulted in cellular ADM concentrations that were 3 orders of magnitude higher than the plasma concentrations at 24 to 48 h after administration.

When the same dose of ADM was administered as a 4 h constant-rate infusion, maximal plasma concentrations were ~60–80 ng $ml^{-1}$. The cellular concentrations showed peak levels of 1100–1200 ng $ml^{-1}$. In case of a 4 h infusion the

**Figure 1** Representative plasma (●) and cellular (×) ADM and plasma (○) adriamycinol concentration time curves after ADM 30 mg $m^{-2}$ administered as bolus injection (a), 4 h (b) and 24 h (c) continuous infusion. Arrows indicate the administration times. Cellular levels refer to peripheral blood cells, shown to correlate well with bone marrow ADM concentrations (see text).

ADM efflux from the cells at the end of the infusion was much less extended compared with bolus injection. In the second, slow ADM disappearance phase, the cellular and plasma ADM curves had similar half-lives to those observed in the case of bolus injection. Constant-rate infusion of 24 h resulted in even lower maximal plasma levels (20 ng ml$^{-1}$) and a slow but continuous increase of cellular ADM concentrations during the infusion period. At the end of the infusion hardly any loss of cellular ADM was observed.

In all three modes of drug administration 1 h after the plasma ADM concentrations had reached negligible values, (<10 ng ml$^{-1}$) almost identical cellular drug concentrations were attained (Figure 1).

### In vitro *cellular ADM concentrations*

Human bone marrow cells were exposed to a range of ADM concentrations for 5 min, 2 h and 24 h. Cellular ADM uptake and release were measured during ADM exposure and subsequent reincubation in drug-free medium ($n=5$). Figure 2 shows an example of the cellular ADM levels after 5 min, 2 h and 24 h exposure to ADM concentrations of 5000, 400 and 40 ng ml$^{-1}$ respectively. The incubation concentrations in this example were chosen to result in similar growth-inhibition of clonogenic cells (80%). It is seen that the plateau concentrations (cellular ADM concentration at reincubation) were comparable. Furthermore the time courses of cellular ADM concentration were essentially the same as observed after *in vivo* bolus injection and short- or long-term infusion respectively (Figure 1). A rapidly attained and high cellular ADM concentration after the short-lasting exposure, was followed by a considerable loss (87–91%) during the two wash steps. During the 2 h and 24 h exposure a more gradual increase of cellular ADM concentrations was followed by a less pronounced loss after the wash steps (27–51% and 12–23% respectively, range of 5 experiments).

**Figure 2** Example of cellular ADM concentrations attained after *in vitro* exposure of bone marrow cells during 5 min to 5 μg ADM ml$^{-1}$ (a), 2 h to 400 ng ADM ml$^{-1}$ (b) and 24 h to 40 ng ADM ml$^{-1}$ (c). Arrows indicate the incubation times. The heights of the arrow indicate the medium ADM concentrations.

### *Cellular ADM concentrations and clonogenicity (CFU-GM)*

In 2 patients clonogenicity was determined before and 1 h after ADM 30 mg m$^{-2}$ push injection. Clonogenicity was depressed from 100% (before) to 79 and 44% after ADM. In 1 patient clonogenicity was determined on 6 consecutive occasions before and after a 4 h infusion of ADM in increasing dosages of 40 to 80 mg m$^{-2}$. Inhibition of clonogenicity at the end of the infusion decreased from 51% (40 mg m$^{-2}$) to 30% (80 mg m$^{-2}$) of the pre-infusion values.

The *in vitro* concentration-response curves for all 3 exposure times are plotted in Figure 3 ($n=5$). The concentration at which 50% inhibition of clonogenic cells occurred (IC50) was calculated from the fitted curves. After 5 min exposure the IC50 was 2.2 μg ml$^{-1}$ ADM. Prolonged exposure resulted in cytotoxicity at lower ADM medium concentrations, the IC50 being 0.35 and 0.05 μg ml$^{-1}$ after 2 h and 24 h respectively. An even lower IC50 of 0.0056 μg ml$^{-1}$ was achieved after continuous exposure during 12 days culturing when ADM had been added to the semi-solid culture medium.

Figure 4 shows the inhibition of clonogenicity as a function of the cellular ADM concentrations, measured at the moment of plating the cells in semi-solid medium. For each point the s.d. ranged between 5 and 25%. The fitted concentration-response curves did not show significant differences.

### Discussion

The first part of the *in vivo* cellular ADM concentration-time curves observed after administration of 30 mg m$^{-2}$ ADM as bolus injection, short-lasting and long-lasting infusions

**Figure 3** Inhibition of clonogenic cells at increasing ADM concentrations for different incubation times. (+) = 5 min incubation, (×) = 2 h incubation, (◇) = 24 h incubation, (□) = continuous exposure. The relative number of colonies is expressed as mean percentage of two duplicate control cultures for each incubation time ($n=5$). S.d. varied from 5–15%. Curves were fitted as described in the text.

**Figure 4** Inhibition of clonogenicity plotted against cellular ADM concentrations expressed in fluorescence units per cell (FU/cell) measured after ADM exposure. (+) = 5 min, (×) = 2 h and (◇) = 24 h exposure. The relative number of colonies is expressed as percentage of two duplicate control cultures for each incubation time ($n=4$). Curves were fitted as described in the text.

appeared essentially different; after bolus injection high peak concentrations were observed, in contrast to long-lasting infusions, showing a gradual increase of cellular ADM. However, the second part of the concentration-time curves, from the moment the plasma concentrations became negligible, the cellular ADM concentrations reached comparable levels and showed similar disappearance rates.

The cellular ADM disappearance curves observed *in vitro* after 5 min, 2 h and 24 h exposure showed similar patterns as observed *in vivo* after a bolus injection, a short- and long-lasting infusion respectively. The shorter the ADM-infusion or the ADM-exposure time *in vitro* the more ADM was accumulated intracellularly in the initial phase, but also more of the initially accumulated ADM was subsequently lost. The net result was comparable ADM concentrations if the same total dose was used (*in vivo*), called 'tightly bound' ADM in contrast to the 'loosely-bound' ADM lost after disappearance of plasma ADM. *In vitro* the 'tightly-bound' ADM concentrations correlated well with the level of inhibition of clonogenic cells. Loss of cellular ADM after reincubation in drug free medium has been described previously (Preisler & Raza, 1984). Although the mechanism behind this observation is speculative, it may be explained by a two compartment model with a fast but reversible cytoplasmic accumulation followed by an irreversible intercalation into DNA at a slower rate (Skovsgaard, 1978).

Both Nguyen-Ngoc *et al.* (1984) and Andersson *et al.* (1982) have concluded from their *in vitro* experiments that the cellular peak-concentrations of anthracyclines obtained after bolus injection, produce a higher cytotoxicity than the lower ADM concentrations after long-lasting infusions. Their conclusions were based on the *in vitro* observation that the ADM dose (product of concentration and exposure time) had to be increased with increasing exposure time. The results from this study indicate however that *in vivo*, administration of the same dose at different infusion rates results in comparable 'tightly-bound' cellular ADM concentrations and comparable inhibition of clonogenic cells. This apparent controversy has to be explained by the translation by the above-mentioned authors of *in vitro* results to the *in vivo* situation, ignoring the totally different exposure conditions in the body (e.g. plasma disappearance curves, protein binding) and in the test tube. The concept of dose in the *in vivo* situation, cannot be considered equivalent to the *in vitro* concentration-time product. However, *in vivo* and *in vitro* comparisons can be made if the cellular ADM concentrations are the same during the experiments.

Bailey-Wood *et al.* (1984) concluded from their *in vitro* observations that the high drug concentrations present in the plasma for short periods of time following rapid i.v. infusion were only weakly cytotoxic and advised long-lasting infusions and low dosages.

In this study we have shown that short-lasting exposure to high concentrations can be just as cytotoxic as long-lasting exposure to lower ADM concentrations, provided that similar cellular ADM concentrations are retained after the elimination of plasma (or medium) ADM. In clinical practice, however, bolus injection in contrast to long-lasting infusions causes more side-effects, such as nausea and vomiting and increased cardiotoxicity.

Preliminary results showed that the concept of cytotoxicity to be correlated directly to the 'tightly-bound' cellular ADM fraction is valid for leukaemic clonogenic cells (data to be published).

'Tightly-bound' cellular ADM levels observed after a total dose of $30\,mg\,m^{-2}$ did not result in complete inhibition of clonogenic cells. Also clonogenicity was not completely depressed in the patient treated with increasing ADM dosages up to $80\,mg\,m^{-2}$. These results indicate that the *in vivo* attained 'tightly-bound' cellular ADM concentrations are not high enough to completely kill clonogenic cells. If these observations are also valid for leukaemic clonogenic cells, therapeutic schemes containing anthracyclines should be reconsidered.

### References

ANDERSSON, B., BERAN, M., PETERSON, C. & TRIBUKAIT, B. (1982). Significance of cellular pharmacokinetics for the cytotoxic effects of daunorubicin. *Cancer Res.*, **42**, 178.

BAILEY-WOOD, R., DALLIMORE, C.M. & WHITTAKER, J.A. (1984). Effect of adriamycin on CFU-GM at plasma concentrations found following therapeutic infusions. *Br. J. Cancer*, **50**, 351.

BENJAMIN, R.S., CHAWLA, S.P., HORTOBAGYI, G.N., EWER, M.S., CARRASCO, C.H. & MACKAY, B. (1985). Adriamycin administration by continuous infusion. *EORTC symposium on continuous infusion chemotherapy*, Brussels, (*abstract*).

DE WITTE, T., KOEKMAN, E., PLAS, A. & 4 others (1982). Enrichment of myeloid clonogenic cells by isopycnic density equilibrium centrifugation in percoll gradients and counterflow centrifugation. *Stem Cells*, **2**, 308.

GARNICK, M.B., WEISS, G.R., STEELE, G.D. & 4 others (1982). Clinical evaluation of long-term continuous infusion of doxorubicin. *Cancer Treat. Rep.*, **67**, 133.

LEGHA, S.S., BENJAMIN, R.S., MACKAY, B. & 6 others (1982). Reduction of doxorubicin cardiotoxicity by prolonged continuous intravenous infusion. *Ann. Int. Med.*, **96**, 133.

LEWIS, J.P., MEYERS, F.J. & TANAKA, L. (1985). Daunomycin administered by continuous infusion is effective in the treatment of acute nonlymphocytic leukaemia. *Br. J. Haematol.*, **61**, 261.

MURRAY, W. (1972). *Numerical methods for unconstrained optimization*. Academic Press: London.

NGUYEN-NGOC, T., VRIGNAUD, P. & ROBERT, J. (1984). Cellular pharmacokinetics of doxorubicin in cultured mouse sarcoma cells originating from autochtonous tumors. *Oncol.*, **41**, 55.

PREISLER, H.D. & RAZA, A. (1984). Uptake of adriamycin by human leukemic cells as measured by flow cytometry. *Med. Oncol. Tumor Pharmacother.*, **1**, 43.

SKOVSGAARD, T. (1978). Carrier-mediated transport of daunorubicin, adriamycin and rubidizone in Ehrlich ascites tumor cells. *Biochem. Pharmacol.*, **27**, 1221.

SPETH, P., LINSSEN, P., DE PAUW, B. & DE WITTE, T. (1986). Cellular pharmacokinetics of daunomycin administered as continuous intravenous infusion in the treatment of acute non-lymphocytic leukaemia. *Br. J. Haematol.*, **63**, 602.

SPETH, P., LINSSEN, P., BOEZEMAN, J., WESSELS, J. & HAANEN, C. (1985). Quantitation of anthracyclines in human hematopoietic cell subpopulations by flow cytometry correlated with high pressure liquid chromatography. *Cytometry*, **6**, 143.

SPETH, P.A.J., LINSSEN, P.C.M., BOEZEMAN, J.B.M., WESSELS, J.M.C. & HAANEN, C. (1986). Rapid quantitative determination of four anthracyclines and their main metabolites in human nucleated hematopoietic cells. *J. Chromatogr.*, **377**, 415.

VERMA, D.S., SPITZER, G. & BERAN, M. (1980). Colony stimulating factor augmentation in human placenta. *Exp. Hematol.*, **8**, 917.